# On the Use of Arsenic in Cancerous Complaints

**Published:** 1815-09

**Authors:** Thomas Sewall


*For the London Medical and Physical Journal.*

### *On the Use of Arsenic in Cancerous Complaints;* by THOMAS SEWALL, M.D.

THE celebrated empiric, Peter Davison, whose pretensions to the cure of cancers have of late excited so much interest in the minds of the community in this part of the country, has recently visited this town. He has here had a large number of applicants labouring under cancerous complaints, some of which were well defined, and had long resisted the ordinary modes of treatment. A number of these he has treated with complete success; and nothing but time is wanting to prove that these cures are radical.

He applies his remedy to the cancer in the form of a plaster, which excites great pain, inflammation, and swelling, and appears to possess the remarkable power of destroying the vitality of the whole of the scirrhus or cancerous part. This soon sloughs off, leaving nothing but a simple ulcer, which is easily healed by the ordinary means.

I have now the pleasure to inform you, that, by a number of experiments, I believe, I have detected the composition of his plaster, and find it to contain *arsenic*, which, I have good reason to suppose, is its only active ingredient.

This is disguised by the admixture of a large proportion of some vegetable powder. The composition is generally mixed with the yolk of an egg, spread on pieces of fine linen proportioned to the size and form of the diseased part, to which it is confined by strips of adhesive plaster extending some way over the sound skin.

I am now using arsenic in cases of cancer in the same form, in which he applies it, and with a degree of success equally flattering.

I prepared

I prepared my plaster with half a drachm of white arsenic levigated and mixed with the yolk of an egg, a little honey, and a sufficient quantity of flour to form the whole into a paste of a proper consistence to be spread on linen. Probably a much smaller proportion of arsenic would have answered equally well.

I am inclined to think this form of applying arsenic to cancers is the most eligible that has hitherto been practised. Its action is here powerful, uniform, and constant, advantages which the powder and solution of this mineral do not combine.

A number of Davison's patients were situated where I had frequent opportunities of inspecting them while under the operation of his medicine, and I have seen nothing but the effect of the external application of arsenic carried to an unexampled extent.

It will at once be recollected, that the use of arsenic in the treatment of cancers is no new practice. It has formed the base of nearly all the quack remedies that have ever been brought before the public. The celebrated cancer powders and washes of Plenket, Guy, Martin, and Pope, were indebted to it for all their activity and success.

But the use of arsenic has not been confined to the hands of quacks: regular physicians have long considered it a valuable acquisition to the materia medica, and a powerful remedy for some of the most formidable diseases.

Dr. Fisher has for many years been in the habit of applying the powder and solutions of this mineral to cancerous and scrofulous ulcers with the most happy effect. In his hands, it has always corrected the discharge of such sores, and often effected a separation of the diseased from the sound part, and thereby performed permanent cures.

The frequent failure of arsenic in the treatment of cancers, while in the hands of regular practitioners, I apprehend, is to be ascribed generally to some defect in the mode of its application, to its indiscriminate use in all cases, to a want of confidence in the patients, but especially to a want of boldness, energy, and perseverance, in the practitioner.

Ere long I hope to be enabled to lay before you a history of a number of well attested cases of cancer cured by arsenic, together with a view of the discriminating marks of such cases as warrant its application.

*Ipswich, December* 16, 1814.

*Note.*—Mr. Peter Davison appears to be acting a part which has already been performed with equal success by hundreds of others of the same fraternity, and his medicine, like like that of his predecessors, will, no doubt, meet with the same fate. The external use of oxide of arsenic, as Dr. Sewall observes, is not a new remedy in cancer. Its power over this complaint, as well as those of a scrofulous nature, has been amply investigated by Hahnmann, le Febure, Justamond, Plenck, Monro, and many others. If these men have considered it as a safe and effectual remedy in this disease, how comes it that its use has been, and is now, confined to the empirics? The physicians and surgeons of the present day cannot, with justice, be accused of timidity in practice; and, if the conclusions of the above-mentioned authors were favourable to the application of arsenic in the treatment of cancer, we doubt not that it would have been adopted with avidity by regular practitioners. But do these authors recommend the adoption, or even speak in civil terms of this mineral poison? On the contrary, Plenck observes, that he has known many unfortunate accidents to arise from its external application. Monro says, he has cured some tumours, which were supposed to have been scrofulous or steatomatous, by it; but that the *true cancerous tumours*, which had been removed by its means, were reproduced, like those that had been extirpated by the knife; and he believes that we have no adequate motive for persisting in the use of so dangerous a remedy. Dr. Monro applied the arsenic in the same way as that described by Dr. Sewall, viz. mixed uniformly with the yolk of eggs. The French authors alluded to, speak of the danger to the system arising from the absorption of arsenic. Brugnatelli, who has seen the bad effects of this substance, emphatically condemns, in his General Pharmacopœia, its use, both internally and externally. The late experiments of Mr. Brodie also prove, that arsenic, when externally applied, at least to a denuded surface, is capable of being absorbed, and of producing in the system the same train of symptoms as when it is taken into the stomach.

In fact, this substance, as a local remedy, does not appear to have any specific power over the disease. Any other caustic, equally powerful, would probably produce the same effects. When the tumour is small, and the neighbouring parts have not participated in the diseased action, arsenic destroys its vitality, the dead part separates, and suppuration and healthy granulations supervene. Where the situation will allow of it, this state of the part may be produced much more expeditiously, and with much less protracted pain, by the knife. In those cases in which the tumour is extensive, for example, extending from the breast to the glands in the axilla, and in those in which considerable ulceration has

2 taken taken place, the use of arsenic, without taking into consideration the possibility of its producing its specific effects on the system, will be accompanied with excessive pain, without being followed by any permanent relief. We say permanent relief, because, in the ulcerated state of this disease, there is little doubt, that the direct application of this caustic will often, at first, lessen the fœtor, correct the discharge, and cause the surface to assume a healthier appearance. Even the size of the ulcer, for a time, gradually diminishes, and the patient has a flattering prospect of full restoration to health. But these good effects are not produced often without expence to the constitution, for they are purchased with great pain and suffering, and symptoms of general irritation appear in the system. The same consequences likewise follow the use of the narcotics, and particularly of the leaves and extract of the *conium maculatum*. In fact, as a palliative, we are inclined to give the preference to the latter. The good effects of both, however, are but temporary; and they only lengthen the duration, and for a time diminish the horrors, by inspiring hope, of this terrible disease.

The celebrity which arsenic has acquired as a remedy in cancer, appears to have arisen from its indiscriminate application to tumours of all descriptions. From the ignorance of the patient, no distinction can be expected; and from the quack, to whom he applies, and whose decision is to be biassed by his interest, no sincerity, presuming on his knowledge, can be hoped. If true to his craft, all indolent tumours, and all old ill conditioned ulcers, form but one class named cancer. He applies his arsenic: the patient, after some weeks of suffering, both mental and corporeal, is cured. The latter, of course, magnifies the extent of his dangers; and the former boasts of his skill and judgment, being well aware that the reputation of curing one cancer, and having one living witness, will outweigh, in the public mind, the unsuccessful treatment of a dozen, who have been consigned to the silent tomb. We do not doubt that the cases which have given celebrity to any cancer-curer, were those which were not cancerous, and which, if cancerous, might have been cured in a shorter time by the use of the knife. How many of the cases in Ipswich, cured by Mr. Peter Davison, were of this description, we know not. Dr. Sewall is a judicious practitioner, and, we presume, will give more time to the consideration of the nature of the cases in question, before he decides on the utility of a medicine which has already been extensively employed, and been found in-

 adequate adequate to the radical cure of genuine cancer. We must confess it surprised us to find, that the village of Ipswich should furnish, not only Mr. Peter Davison with "a great number of cases," but Dr. Sewall himself with "several cases," of cancer, when the disease is so rarely seen in Boston.—EDITORS. *New-England Journal.*

---

### COLLECTANEA MEDICA,

